# Primary School Pupils' Perceptions and Experiences of Wearable Technologies

**DOI:** 10.1111/josh.13509

**Published:** 2024-11-06

**Authors:** Georgina K Wort, Gareth Wiltshire, Simon Sebire, Oliver Peacock, Dylan Thompson

**Affiliations:** ^1^ Centre for Motivation and Health Behaviour Change, Department for Health University of Bath Bath BA2 7AY UK; ^2^ School of Sport, Exercise, and Health Sciences, Loughborough University Loughborough LE11 3TU UK; ^3^ School for Policy Studies, University of Bristol Bristol BS8 1QU UK; ^4^ Department for Health University of Bath Bath BA2 7AY UK; ^5^ Department for Health University of Bath Bath BA2 7AY UK

**Keywords:** physical activity, wearable technology, schools, children

## Abstract

**BACKGROUND:**

Wearable technologies offer new opportunities to address in‐school physical inactivity. However, children are often excluded from discussing issues which directly impact them, including the use of wearable technologies in a school setting. Thus, the aim of this study is to understand primary school pupils' experiences and perceptions of using wearable physical activity monitoring technologies within schools.

**METHODS:**

Nine semi‐structured focus groups were conducted with 41 Year 5 and 6 pupils (19 girls, 22 boys) from 5 primary schools in the South West of England. Focus group transcripts were analyzed using a reflective thematic approach.

**RESULTS:**

Pupils made valuable contributions to discussions around wearable technologies, considering both advantages and disadvantages. Most importantly, when discussing use in schools, pupils were mindful that while they wanted to see their own activity and saw benefit in teachers knowing their activity, they felt comparisons in classes could lead to negative emotions for some pupils.

**CONCLUSION:**

The findings from this study can contribute to a more detailed understanding of pupils' perspectives, which can help inform school‐based interventions which aim to address physical inactivity and associated inequalities. Instead of using wearable devices for individual pupil ownership, schools should access data‐insights with the intention of changing school practices.

The wearable technology industry continues to grow, and this offers new opportunities to address issues such as physical inactivity. Devices are increasingly used to monitor physical activity as part of interventions^1^, ^2^ and providing participants with devices to self‐monitor physical activity has been shown to be effective in improving physical activity in adults^3^, ^4^ and children.^5^, ^6^, ^7^ School‐based interventions, which have used feedback from wearable technologies, have shown positive physical activity outcomes.^8^, ^9^, ^10^, ^11^ Outside of a research context, these technologies are increasingly being used at home and in schools, and it is reasonable to assume that children from more affluent backgrounds, or with families more engaged in physical activity, are more likely to have access to these devices. However, despite suggestions of the promise and effectiveness of such technologies for improving physical activity, it has also been reported that digital interventions targeting physical activity are less effective for those from lower socio‐economic backgrounds^1^ and Evans and colleagues^12^ found the use of Fitbit devices did not increase physical activity in a low‐income group within school settings. Therefore, the use of physical activity wearables in school‐based interventions warrants consideration as, if they are not carefully managed, they have the potential to further exacerbate inequalities in physical activity.^13^, ^14^

Children's voices are often excluded from issues which directly impact them, with misconceptions that they lack the capacity for meaningful discussions.^15^, ^16^, ^17^, ^18^ However, at the same time, it is acknowledged that researchers should use bottom‐up approaches, understanding the perspectives and knowledge of individuals at the center of the phenomenon of interest.^19^, ^20^ Research has demonstrated that teachers believe the integration of physical activity wearables within school settings is feasible and acceptable,^21^, ^22^, ^23^, ^24^ yet adolescents have reported less favorable opinions.^25^ Concerns have been raised that wearable physical activity monitors could contribute to the development of disordered eating, excessive physical activity, or bodily dissatisfaction.^26^, ^27^, ^28^, ^29^, ^30^, ^31^ However, this research has often used physical activity monitoring alongside diet and calorie tracking and more research is needed to understand children's relationship with wearable technologies which are used to monitor physical activity alone. While there have been calls to teach children and youth how to critically engage with digital health technologies,^32^ they are often left out of the conversation and their views and experiences of wearable technologies in schools is underreported. It has been demonstrated that children aged 9 to 12 appreciate and enjoy being involved in decision making^33^ and that adolescents make valuable contributions to conversations about addressing physical inactivity and the associated inequalities.^16^, ^34^, ^35^ However, this approach has not been utilized in primary‐school children, particularly in the context of wearable technologies. To our knowledge, no studies have sought to understand children's views on physical activity wearable technologies within primary schools.

The aim of this study is to understand primary school pupils' experiences and perceptions of the utilization of wearable physical activity technologies in school environments.

## METHODOLOGY

### Participants

Schools were invited to take part by contacting PE leads or headteachers. Focus groups were run with Year 5 and 6 pupils (aged 9‐11) across 5 schools based in the South‐West of England, with between 1 and 2 focus groups in each school. Details of participating schools are in Table 1. The aim was to gain a broad representation and include schools which represented a range of rurality, size, and socio‐economic status of the school community (using free school meal percentage as a proxy). Furthermore, we included schools who had never used physical activity wearable technologies and schools who had been involved in research which had used class‐based wearable technologies. The aim was to include a range of pupils, including individuals who had never owned a device, had previously personally owned a device, and/or had the experience of using physical activity devices within schools. Approximately half of the pupils had some experience of using wearable technologies within the school environment, with 3 out of the 5 schools having previously used physical activity wearable technologies in a research capacity.

**Table 1:** Focus Group Information

Focus Group (FG) Number	School Number	Pupils	FSM %	OFSTED Rating	School Used Wearables
1	1	3 (2 girls, 1 boy)	4.31%	Good	Y
2		4 (1 girl, 3 boys)			Y
3	2	5 (2 girls, 3 boys)	7.69%	Good	N
4		4 (2 girl, 2 boys)			N
5	3	5 (2 girls, 3 boys)	17.23%	Good	Y
6		4 (1 girls, 3 boys)			Y
7	4	7 (3 girls, 4 boys)	8.08%	Outstanding	N
8	5	5 (2 girls, 3 boys)	10.75%	Good	Y
9		4 (4 girls)			Y

Information packs and consent/assent forms were sent home to all pupils in Year 5/6. Forty‐one pupils received parental/guardian consent, and 1 withdrew during a focus group. In consultation with class teacher(s), 3‐7 pupils for each focus group were selected to include a range of different voices, opinions, “friendship groups,” physical capabilities, physical activity preferences, and experiences within school. This purposive sampling strategy was used because teachers are well placed to understand their pupils' behaviors and can provide valuable insights into structuring focus groups to facilitate a mix of pupils' opinions regarding physical activity. Therefore, focus groups contained between 3 and 7 pupils aged between 9 and 11.

### Procedure

The Consolidated Criteria for Reporting Qualitative research was followed to report the study design and analysis of findings and is included in the supplementary information.^36^ Focus groups have previously been shown to be an effective method for gaining insight regarding the thoughts and perspectives of children.^37^, ^38^

The focus group questions were piloted beforehand to check that questions were easily understood and appropriate for the age group. Focus groups were held within each school with some focus groups run during breaktimes, PSHE, or classroom time based on teacher preferences. Focus groups were conducted in a quiet classroom with the chairs arranged in a circle/horseshoe format. The aim was to create a comfortable environment with which the children were familiar. A school staff member was close by, but not in the same room. When children arrived, they were encouraged to wear a name sticker and complete a 10‐point Likert‐scale question to self‐identify how much they engaged in, and liked, physical activity. Children's average self‐selected physical activity score was 8 (SD 1.6), where 1 was the least active, and 10 the most active. Scores ranged from 5 to 10, demonstrating that pupils in the sample viewed themselves more active than average. There was no meaningful difference between boys and girls.

**Figure 1:**
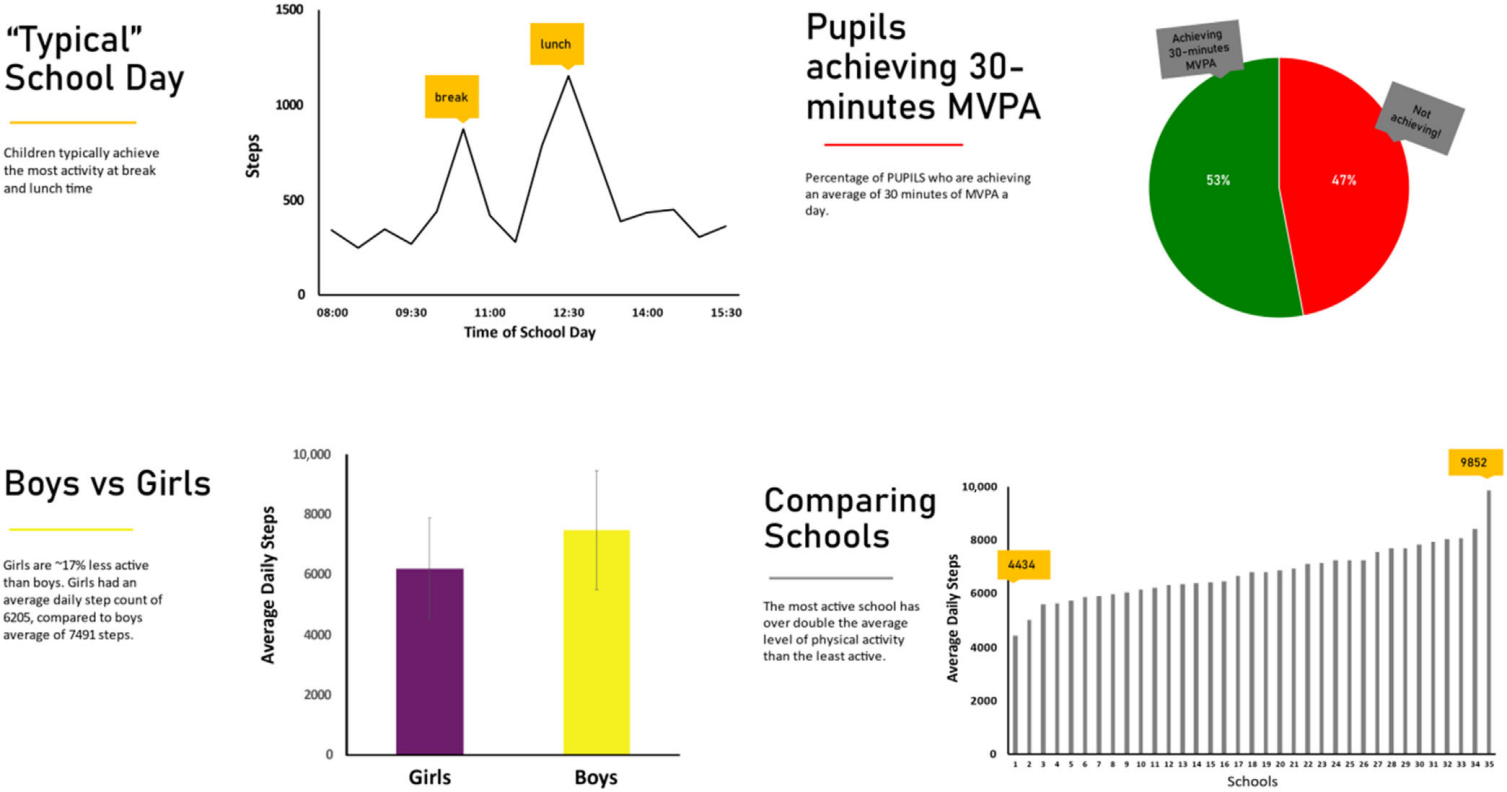
Graphs Shown to Pupils' During Focus Groups, the Data Populating the Graphs Was Obtained From Physical Activity Wearable Technologies Used in Primary Schools

The researcher then introduced themselves, explained the purpose of the focus group, and reminded pupils about confidentiality and anonymity and the ability to leave at any point. It was made clear that any information discussed would not leave the room and was only for the purpose of the researcher. The pupils were told that the researcher was interested in finding ways to encourage children to be active and the focus groups were to understand their views about physical activity, its related barriers, and facilitators, as well as their opinions of wearable technologies. The aim was to allow pupils to articulate their own experiences and opinions. An ice‐breaker activity was then used to help pupils feel more comfortable contributing whereby children were asked to introduce themselves and tell the group about their favorite activity. Throughout the focus groups pupils were reminded that there were no right or wrong answers and that it was expected that opinions would differ from each other, and at points they were asked whether they agreed with statements made by other pupils. Pupils were shown graphs, (eg, Figure 1), which used data previously derived from physical activity wearable technologies in primary schools to stimulate conversation.^21^ Pupils were asked about their experiences using wearable technologies, for example, did any of them personally use devices, or did their school use any technologies relating to physical activity. They were also asked about how they might feel about firstly receiving data about how active they've been, and secondly having this shared within their class. The researcher prompted pupils to discuss how others might feel, for example, did they think everyone would feel or respond in the same way? A focus group interview guide was used to facilitate discussions (Table 2). Focus groups lasted 39 minutes on average (range = 31 to 45 minutes).

**Table 2:** Focus Group Question Structure

Focus Area	Examples of Questions
Introduction	Explain why the pupils are here‐ what is the purpose of the focus groups Introduce myself—PhD researcher at the University of Bath. My research aims are to find out ways to encourage primary school children to be more active Set intentions, confidentiality, and anonymity as well as right to withdraw
Children's activity preferences	What are your favorite things to do? What do you enjoy doing out of school, what do you do at weekends or after school? What do you enjoy doing most in school? What do you play at break and lunchtimes? What opportunities do you think you have to be active? What do you think of P.E.? What physical activity do you do? What do you think counts as physical activity?
Views on physical activity	Do you think you're more active in school or outside of school? In school when do you think you're most active? What do you think prevents you being active? What do you think encourages you to be active? Why do you think some children are more active than others? Do you think anything could, or should, be done to encourage those who are less active?
Views on data	These graphs will contain generate physical activity data, as obtained from wearable technologies to depict differences in individual pupils' physical activity within the school day, gender differences, differences between schools and proportion of children active Do you understand these graphs? What do you make of knowing that? Why do you think there are such differences between pupils just within the school day? Are you surprised at the differences between boys and girls? Do you think it should be that way? Why do you think there are differences between pupils in different schools?
Views on technologies	Have you ever used Fitbits, Garmins, or anything similar? Have any of your friends used Fitbits or anything similar? What were your or their experiences? Do you think individuals have equal access to these devices? What do you think about introducing these types of technologies to schools? What do you think of the introduction of activity bands to schools which have no screen on the band, are scanned in and monitored by teachers? How would you like them to be used (if you did)? What do you think the positives could be? Are there any negatives you can think of, or any concerns you have? Do you think children would equally enjoy using them? How would you respond to seeing data on your physical activity? How do you think other children might respond? Are some children more competitive than others? What do you think it would mean for the use of these types of devices?

### Data Analysis

Audio recordings were transcribed verbatim. Notes were made throughout, and the researcher reflected on underlying meanings and how pupils interpreted their experiences.^39^, ^40^, ^41^ NVivo 12 was used to facilitate the analysis, the coding was conducted by GKW, and codes and themes were discussed between GKW and GW, revised several times until they were reflective of the data and address the research aims. While questions were asked regarding pupils' physical activity in schools, the focus of this study is on pupils' views and experiences of physical activity wearable technologies.

## FINDINGS

Four themes were developed from the data; owning and using devices, advantages of wearable technology, concerns about wearable technology, and implementation in schools.

### Owning and Using Devices

Children highlighted that wearable technologies were already being used in schools; however, some children felt there was unequal engagement. For example, “*some people have digital watches, but some people don't, and the ones that don't are often the less sporty people*.” (Girl, FG1). However, there was disagreement within this, with another girl believing “*it doesn't matter if you're into sports or not into sports, sometimes you just want it [wearable device] to see how many steps you can do*.” (FG1).

Many of the children in this study owned a device themselves, however, as the following quotes illustrate, they often forgot to charge devices, wore them intermittently, experienced discomfort/irritation or devices breaking.

“I forget to charge mine” *(Boy, FG7*).

“I do have a Fitbit, but the strap broke” *(Boy, FG8*).

   I had to stop wearing it because it was wearing into my skin, and I had a really big rash *(Boy, FG3*).

Children highlighted practical considerations for devices, such as the esthetics, personalization, comfort, as well as cost being a barrier. Children believed that an element of responsibility was needed for children to own wearable technologies, and that younger children would not properly look after devices as this quote highlights: “*I also think it would just be for the older years because if a reception [pupil] had it they'd probably break it*.” (Boy, FG4). Some pupils also alluded to concerns over the accuracy and reliability of devices, including the impact that wearing a device had on their behavior: “e*veryone in my class was walking slow, but moving their hands fast so they could get more steps*.” (Boy, FG2). There was the perception that “*some people get [devices] and then they don't use them properly*” (Boy, FG4), for example messaging or playing games, rather than using for physical activity monitoring. This girl said she had heard “*watches were confiscated in their school because people weren't using them for their exercise, they were using them to play games on*.” (Girl, FG1) and another pupil shared that “*somebody in our class they got it [a wearable device], they just don't wear it to school anymore because it's proved too much of a distraction, I guess. But he got in taken away 5 times*.” (Boy, FG5).

Pupils were interested in the additional features of devices, rather than just physical activity monitoring. For example, they discussed the messaging features (which often caused distractions), ability to play music or take photos, and safety features. They highlighted that the safety features could potentially be used for benefit or harm, with 1 boy sharing a story of his father using it to check they had arrived when traveling independently: “*on the way to school I have a safety check, so if I don't turn my safety check off within half an hour then it sends out emergency sharing, and then my parents will be like ‘oh he might be in trouble’, or I might just have forgotten to turn it off*.” However, he highlighted there could be negative implications if his “*dad lost his phone and someone else got it and then they were tracking me*.” (Boy, FG5).

### Advantages of Wearable Technologies

Several pupils highlighted that they found that devices, with the ability to set goals, and receive positive feedback motivated them to be active. For example, pupils said “*It kind of got me moving a bit more*.” (Boy, FG2) or “*I think it's quite helpful, because I had this Fitbit and when you do 10,000 steps this disco ball comes down*.” (Boy, FG4). One girl said, her device “*tells me what steps [I've done], and the next day I try and beat what I've done*.” (Girl, FG1), another “*found it motivating*” (Boy, FG1). Some pupils felt that for “*those who weren't as active, it might encourage them*” (Girl, FG9) with other suggestions that extrinsic rewards, like in‐class prizes, could be used alongside technologies to further motivate inactive children. Additionally, pupils commented on the value of devices monitoring sleep as well as physical activity; “*I use mine a lot just to check my steps, I enjoy looking at them… I can see how many kilometres or steps I've walked. And it also shows me how much sleep I've had on it*.” (Girl, FG1).

### Concerns about Wearable Technologies

While many were positive about the use of wearable technologies, not all pupils liked the idea of using devices to measure physical activity. As the following quote illustrates, some disliked the idea of being measured, or having a judgment of health placed on them:

“I don't really want one, because I want to be my own judge of how I'm healthy. And I don't really want a Fitbit to judge that for me.” *(Girl, FG3)*.

“But I just don't like the idea of having something that can measure what I'm doing on me.” *(Girl, FG3)*.

One girl alluded to the potential for negative thoughts or stating that “*because you could see how many calories you were burning, she* [her sister] *was trying to get a certain goal every single day*.” (FG4), something which she did not view positively. Some pupils also highlighted that feedback had the potential to cause negative emotions:

Girl: sometimes when you don't get much steps you can feel ashamed of yourself

Boy: yeah, you feel bad about yourself *(FG2)*

Pupils talked more broadly about technology and potential concerns, such as fears over privacy, addiction, prolonged screen‐time use and the impact on social skills.

Some people would be worried, like they were tracking them. *(Boy, FG3)*

“Yeah it was addictive. It could disimprove your social skills because you'll always be looking at that.” *(Boy, FG2*)

“I think having a Fitbit, when you've got it it's good, but it's bad. It's good because you can check what you need to do, get more steps, get a better active time, [know] how much your sleep is, but then it's bad for you because you're looking a screen and if you think about it, however much you're looking at that screen, it's hurting your eyes, so it's good to have one for average steps, getting more.” *(Boy, FG7*)

### Implementation in Schools

When considering the use of wearable physical activity technologies in schools pupils believed that devices could be a distraction and shared examples of other pupils within their class, as highlighted by the conversation between 2 boys: “*Boy: they can also be a bit distracting in class…Boy: Sam has got his band confiscated quite a lot*” (FG3). Pupils also believed that due to their novelty, the use of wearable technologies in schools to measure activity needed to be fair and available to all pupils. One girl thought schools “*should bring really, really cheap Fitbits into schools so everyone could keep track of their steps*” (Girl, FG9). Another felt that “*if all the other classes had one [wearable device] and some other classes didn't, I'd feel a bit disappointed because they've had the opportunity and we haven't*” (Girl, FG4). When discussing the sharing of data in classes, there were mixed feelings. Some children highlighted that they “*like seeing how much exercise [they've] done a day*” (Girl, FG2) and that they would enjoy the competitive nature of comparing step counts: “*It was competitive wasn't it, everyone was like, I'm gonna get more steps than you*.” (Boy, FG6). For other pupils' comparisons of individual activity levels could cause negative emotions: “*if they realise how much somebody else has done, they would be a bit embarrassed to see that they've done not as good as the other people*” (Girl, FG1). And as the following dialog illustrates there were conflicting views: “*Boy: That's what most people want, they want to be competitive. Girl: I don't think that would make everybody happy*” (FG8). Another girl said, “*I wouldn't like the idea of them being displayed because then I feel like people would just point out how many less* [steps] *you've got*.” (FG4). Instead, a girl thought it would “*be helpful if the teachers could show it with us individually*” (Girl, FG7). They thought that teachers could use devices to understand physical activity within their class, with the potential to encourage activity at a class level if it was low. A girl suggested “*it's good for teachers to see it, because if everyone's steps are really low, the teacher could see, and you could go into the playground and just run around*” (FG7).

## DISCUSSION

This study demonstrates that primary school pupils are capable of valuably contributing to discussions about the use of physical activity wearable technologies within schools. Children's voices are often underrepresented in research^42^ and these novel findings provide insight into children's views on the use of such technologies. The findings highlight practical considerations which can be used for future intervention design and development, as well as important considerations over the introduction of wearable technologies into school practices.

Children articulated a range of positive and negative views about wearable technologies, and they were mindful about how other children may respond to having their physical activity data shared. Children highlighted that, while feedback could be useful to inform teachers of their classes' physical activity, this data should perhaps not be shared in front of other pupils in the class due to concerns over negative emotional reactions. Therefore, the way in which data are fed back is important and requires consideration. It is reasonable that negative physical activity feedback, alongside peer comparisons, particularly with young children, would cause similar emotional responses, decrease motivation and self‐efficacy, in turn harming physical activity engagement.^43^, ^44^ As the pupils in this study alluded to, having pupil‐level physical activity values compared among a school class may be most detrimental to the least active pupils.

Pupils suggested that, instead of sharing data with the whole class, receiving individual feedback would be appropriate, highlighting the motivating effects of goal setting. Indeed, goal setting through wearable technologies has been reported to be a useful tool in the wider population, with children, and adolescents.^45^, ^46^, ^47^ However, there have been criticisms of physical activity wearable technologies previously relying too heavily on individual behavior change^2^ and there are other ways wearable technologies can be utilized. As suggested by pupils in this study, data could be used to inform teachers of their classes' physical activity behaviors with the ability to intervene to address periods of inactivity. Indeed, interventions in school settings which have used pupils' physical activity data to inform teachers have been reported to be effective.^8^, ^10^, ^48^, ^49^ However, how these data‐driven insights are managed is important, as the pupils in this study highlight. Children engage in physical activity for enjoyment,^50^ and those who have higher physical competencies are more likely to enjoy physical activity and have sustained engagement.^51^ While these technologies do not measure physical competency, children with low physical activity or proxy measures such as step counts, may then perceive themselves as less competent. It could be argued that, instead of concerning children with measures of activity, we should make sure schools focus on fostering fun and positive experiences to support longer‐term engagement in physical activity. This would mean that instead of using wearable devices with the intention of pupils taking individual ownership of their physical activity measures, teachers and schools should access data‐insights with the intention of changing school practices, addressing areas of inactivity throughout the school day, and delivering additional provision to support those least active.

Previous research has demonstrated the important role teachers can play in either facilitating or inhibiting pupils' physical activity.^50^, ^52^, ^53^ Involving those who have a clear understanding of the school environment and individual pupil challenges would allow a pragmatic approach, which can be adapted for varied contexts.^54^ Teachers are well placed to understand pupils' needs and individual differences, navigating issues like social comparisons such as the sharing of academic grades. However, it is also reasonable that they may implement well‐meaning strategies, such as individual step competition, with unintended negative consequences. Pupils alluded to data privacy and security concerns through comments relating to fears over monitoring and safety features. However, children often have limited autonomy in traditional schooling, which is important particularly as it has been reported that teachers lack consideration over who is able to access data, with authors raising concerns over data privacy rights.^14^ As demonstrated in this study, consulting with children is important in understanding how strategies or changes in school practices may be received by pupils. This type of approach provides an opportunity for teachers to engage in discussions regarding data ownership with children, for whom this will be a notable consideration throughout their lives.

There are important issues that warrant careful consideration, particularly as technologies become more pervasive in schools. These types of technologies provide accessible opportunities for supporting children's physical activity. Implementing these physical activity devices in schools, if carefully managed, could be used to address some of the disparities in physical activity, reduce the digital divide and have the potential to support children's academic learning and data literacy skills.^55^, ^56^ Currently, those from more affluent backgrounds, or with parents more engaged in physical activity are likely to have access to physical activity wearable technologies. Despite the cost of commercial devices reducing, they are still not accessible for all children, either due to financial reasons, or a lack of parental support. Schools could be a place where children have equal provision and access to physical activity devices, giving all pupils the opportunity to understand their own physical activity behaviors.

To summarize, while wearable technologies offer new solutions and novel approaches to support pupils' physical activity, careful consideration should be given to how they are implemented in schools. Consulting with children when introducing new approaches is important to mitigate against any unintended negative consequences.

### Strengths and Limitations

This study provides novel insights into primary school pupils' views on introducing wearable technologies, and the accompanying data, in schools. Children within this study had a range of prior experiences with wearable technologies; the sample included those who had never used wearable devices, those who had used personal physical activity devices, and others who had used devices which had been implemented within classrooms as part of research. Therefore, the pupils included in this study had diverse experiences with technology. Importantly this work highlights the valuable contributions children can make when discussing complex issues. Children's voices should be included more when researching issues which directly impact them.

Focus groups can provide different perspectives and provide rich data from the interactions between individuals who have different perspectives. It is reasonable that children felt inclined to answer questions a certain way, either to conform with the group norm, or due to social desirability. For example, pupils self‐reported they were more active than average, perhaps wishing their peers or the researcher to view them this way. It may have been due to the nature of recruitment; a limited number of parent/carers provided consent and it is plausible those from more active families were more likely to be provided with consent and have a wish to discuss these topics. The researcher may have received different responses if pupils were unaware of the focus group purpose, for example, knowing that the lead researcher was interested in physical activity in schools may have led to social desirability bias.

While the OFSTED ratings of schools in this sample were representative,^57^ the average FSM percentage (a proxy indicator for deprivation) of schools across the UK was 22.5 in 2022,^58^ the schools in this sample had fewer pupils eligible for FSM, and lacked representation from marginalized communities, children with disabilities, and probably included children who were more active than average. Future research should aim to include more diverse groups, particularly when using this approach to inform intervention design.

## IMPLICATIONS FOR SCHOOL HEALTH POLICY, PRACTICE, AND EQUITY

Schools are a critical setting which can help children to become and stay active. There have been calls to use whole school policy approaches to embed physical activity. Technology is likely to play an increasingly important role in this and findings demonstrates that primary school pupils can make valuable contributions to discussions about the introduction of physical activity wearable technologies within schools. The findings contribute to a more detailed understanding of pupils' perspectives, which can help inform intervention design, school policy and practice, which aims to address physical inactivity and the associated inequalities.

### Conclusion

The findings from this study contribute to a more detailed understanding of pupils' perspectives, which can help inform school‐based interventions which aim to address physical inactivity and associated inequalities. Instead of using wearable devices for individual pupil ownership, schools should access data‐insights with the intention of changing school practices.

### Human Subjects Approval Statement

Ethical Approval was obtained from University of Bath Research Ethics Approval Committee for Health, EP 20/21091. It was made clear at the beginning of each focus group that children did not have to participate, and that they could leave at any point. Furthermore, the researcher ensured that children did not feel pressured to contribute to discussions. All pupils who received parental/guardian consent were involved in focus groups.

### Conflict of interest

The authors declare no conflicts of interest.

## Supporting information


**Table S1.** The Consolidated Criteria for Reporting Qualitative research checklist table.

## References

[1] Western MJ , Armstrong MEG , Islam I , Morgan K , Jones UF , Kelson MJ . The effectiveness of digital interventions for increasing physical activity in individuals of low socioeconomic status: a systematic review and meta‐analysis. Int J Behav Nutr Phy. 2021;18(1):148. 10.1186/s12966-021-01218-4. PMC8576797 34753490

[2] World Health Organization . Be he@lthy, be mobile: a handbook on how to implement mobile health for physical activity; 2021.

[3] Laranjo L , Ding D , Heleno B , et al. Do smartphone applications and activity trackers increase physical activity in adults? Systematic review, meta‐analysis and metaregression. Br J Sport Med. 2021;55(8):422‐432. 10.1136/bjsports-2020-102892. 33355160

[4] Larsen RT , Wagner V , Korfitsen CB , et al. Effectiveness of physical activity monitors in adults: systematic review and meta‐analysis. BMJ. 2022;376:e068047. 10.1136/bmj-2021-068047. 35082116 PMC8791066

[5] Ridgers ND , McNarry MA , Mackintosh KA . Feasibility and effectiveness of using wearable activity trackers in youth: a systematic review. JMIR Mhealth Uhealth. 2016;4(4):e129. 10.2196/mhealth.6540. 27881359 PMC5143467

[6] Böhm B , Karwiese SD , Böhm H , Oberhoffer R . Effects of mobile health including wearable activity trackers to increase physical activity outcomes among healthy children and adolescents: systematic review. JMIR Mhealth Uhealth. 2019;7(4):e8298. 10.2196/mhealth.8298. 31038460 PMC6658241

[7] Müller J , Hoch A‐M , Zoller V , Oberhoffer R . Feasibility of physical activity assessment with wearable devices in children aged 4–10 years—a pilot study. Front Pediatr. 2018;6:5. 10.3389/fped.2018.00005. 29435438 PMC5790770

[8] Casado‐Robles C , Viciana J , Guijarro‐Romero S , Mayorga‐Vega D . Effects of consumer‐wearable activity tracker‐based programs on objectively measured daily physical activity and sedentary behavior among school‐aged children: a systematic review and meta‐analysis. Sports Med Open. 2022;8(1):18. 10.1186/s40798-021-00407-6. 35099630 PMC8804065

[9] Morris JL , Daly‐Smith A , Defeyter MA , et al. A pedometer‐based physically active learning intervention: the importance of using preintervention physical activity categories to assess effectiveness. Pediatr Exerc Sci. 2019;31(3):356‐362. 10.1123/pes.2018-0128. 30612529

[10] Hodgin KL , von Klinggraeff L , Dauenhauer B , et al. Effects of sharing data with teachers on student physical activity and sedentary behavior in the classroom. J Phys Act Health. 2020;17:585‐591. 10.1123/jpah.2018-0711. 32335524

[11] Hartwig TB , del Pozo‐Cruz B , White RL , et al. A monitoring system to provide feedback on student physical activity during physical education lessons. Scand J Med Sci Sports. 2019;29(9):1305‐1312. 10.1111/sms.13438. 31033042

[12] Evans EW , Abrantes AM , Chen E , Jelalian E . Using novel technology within a school‐based setting to increase physical activity: a pilot study in school‐age children from a low‐income, urban community. Biomed Res Int. 2017;2017:4271483. 10.1155/2017/4271483. 29670894 PMC5833882

[13] Koorts H , Salmon J , Timperio A , et al. Translatability of a wearable technology intervention to increase adolescent physical activity: mixed methods implementation evaluation. J Med Internet Res. 2020;22(8):e13573. 10.2196/13573. 32763872 PMC7442941

[14] Lupton D . ‘Honestly no, I've never looked at it’: teachers' understandings and practices related to students' personal data in digitised health and physical education. Learn Media Technol. 2021;46:281‐293. 10.1080/17439884.2021.1896541.

[15] Darbyshire P , MacDougall C , Schiller W . Multiple methods in qualitative research with children: more insight or just more? Qual Res. 2005;5(4):417‐436. 10.1177/1468794105056921.

[16] Tannehill D , MacPhail A , Walsh J , Woods C . What young people say about physical activity: the children's sport participation and physical activity (CSPPA) study. Sport Educ Soc. 2015;20(4):442‐462. 10.1080/13573322.2013.784863.

[17] Horowitz JA , Vessey JA , Carlson KL , Bradley JF , Montoya C , McCullough B . Conducting school‐based focus groups: lessons learned from the CATS project. J Pediatr Nurs. 2003;18(5):321‐331. 10.1016/S0882-5963(03)00104-0. 14569580

[18] Kellett M . Empowering children and young people as researchers: overcoming barriers and building capacity. Child Indic Res. 2011;4(2):205‐219. 10.1007/s12187-010-9103-1.

[19] Rutter H , Cavill N , Bauman A , Bull F . Systems approaches to global and national physical activity plans. Bull World Health Organ. 2019;97(2):162‐165. 30728623 10.2471/BLT.18.220533 PMC6357559

[20] Davies A , Mbete B , Fegan G , Molyneux S , Kinyanjui S . Seeing ‘with my own eyes’: strengthening interactions between researchers and schools. IDS Bull. 2012;43(5):61‐67.

[21] Wort GK , Wiltshire G , Peacock O , Sebire S , Daly‐Smith A , Thompson D . Teachers' perspectives on the acceptability and feasibility of wearable technology to inform school‐based physical activity practices. Front Sports Act Living. 2021;3:777105. 10.3389/fspor.2021.777105. 34870198 PMC8636981

[22] Almusawi HA , Durugbo CM , Bugawa AM . Innovation in physical education: teachers' perspectives on readiness for wearable technology integration. Comput Educ. 2021;167:104185. 10.1016/j.compedu.2021.104185.

[23] Creaser AV , Frazer MT , Costa S , Bingham DD , Clemes SA . The use of wearable activity trackers in schools to promote child and adolescent physical activity: a descriptive content analysis of school staff's perspectives. Int J Environ Res Public Health. 2022;19(21):14067. 36360944 10.3390/ijerph192114067 PMC9654652

[24] Marttinen R , Landi D , Fredrick RN , Silverman S . Wearable digital technology in PE: advantages, barriers, and teachers' ideologies. J Teach Phys Educ. 2020;39(2):227‐235. 10.1123/jtpe.2018-0240.

[25] Goodyear VA , Kerner C , Quennerstedt M . Young people's uses of wearable healthy lifestyle technologies; surveillance, self‐surveillance and resistance. Sport Educ Soc. 2019;24(3):212‐225.

[26] Gittus M , Fuller‐Tyszkiewicz M , Brown HE , et al. Are Fitbits implicated in body image concerns and disordered eating in women? Health Psychol. 2020;39(10):900‐904. 32406725 10.1037/hea0000881

[27] Brytek‐Matera A , Pardini S , Szubert J , Novara C . Orthorexia nervosa and disordered eating attitudes, self‐esteem and physical activity among young adults. Nutrients. 2022;14(6):1289. 35334945 10.3390/nu14061289 PMC8948728

[28] Simpson CC , Mazzeo SE . Calorie counting and fitness tracking technology: associations with eating disorder symptomatology. Eat Behav. 2017;26:89‐92. 10.1016/j.eatbeh.2017.02.002. 28214452

[29] Hahn SL , Hazzard VM , Loth KA , Larson N , Klein L , Neumark‐Sztainer D . Using apps to self‐monitor diet and physical activity is linked to greater use of disordered eating behaviors among emerging adults. Prev Med. 2022;155:106967. 10.1016/j.ypmed.2022.106967. 35065981 PMC8832499

[30] Plateau CR , Bone S , Lanning E , Meyer C . Monitoring eating and activity: links with disordered eating, compulsive exercise, and general wellbeing among young adults. Int J Eat Disord. 2018;51(11):1270‐1276. 10.1002/eat.22966. 30508261

[31] Harrison AN , Rocke KD , James Bateman C , Bateman A , Chang SM . Physical activity and disordered eating behaviours: are Caribbean adolescents at risk? Int J Psychol. 2022;57(2):218‐226. 10.1002/ijop.12802. 34398467

[32] Rich E , Miah A . Mobile, wearable and ingestible health technologies: towards a critical research agenda. Health Sociol Rev. 2017;26(1):84‐97.

[33] Anselma M , Chinapaw M , Altenburg T . “Not only adults can make good decisions, we as children can do that as well” evaluating the process of the youth‐led participatory action research ‘kids in action’. Int J Environ Res Public Health. 2020;17(2):625. 31963706 10.3390/ijerph17020625 PMC7014142

[34] Cowley ES , Watson PM , Foweather L , et al. “Girls aren't meant to exercise”: perceived influences on physical activity among adolescent girls—the HERizon project. Children. 2021;8(1):31. 33430413 10.3390/children8010031 PMC7827342

[35] Wiltshire G , Lee J , Evans J . ‘You don't want to stand out as the bigger one’: exploring how PE and school sport participation is influenced by pupils and their peers. Phys Educ Sport Pedagog. 2017;22(5):548‐561. 10.1080/17408989.2017.1294673.

[36] Tong A , Sainsbury P , Craig J . Consolidated criteria for reporting qualitative research (COREQ): a 32‐item checklist for interviews and focus groups. International J Qual Health Care. 2007;19(6):349‐357. 10.1093/intqhc/mzm042. 17872937

[37] Sims T . Piloting the focus group method: preparing for a participatory design study with children and young people; 2017. Available at: https://methods.sagepub.com/case/focus‐group‐method‐preparing‐participatory‐design‐children‐young‐people. Accessed June 30, 2023.

[38] Gibson F . Conducting focus groups with children and young people: strategies for success. J Res Nurs. 2007;12(5):473‐483. 10.1177/1744987107079791.

[39] Wiltshire G , Ronkainen N . A realist approach to thematic analysis: making sense of qualitative data through experiential, inferential and dispositional themes. J Crit Realism. 2021;20:159‐180. 10.1080/14767430.2021.1894909.

[40] Braun V , Clarke V . Reflecting on reflexive thematic analysis. Qual Res Sport Exerc Health. 2019;11(4):589‐597. 10.1080/2159676x.2019.1628806.

[41] Braun V , Clarke V . Thematic Analysis: A Practical Guide. Los Angeles, CA: Sage; 2021.

[42] Hill M . Children's voices on ways of having a voice: Children's and young people's perspectives on methods used in research and consultation. Childhood. 2006;13(1):69‐89.

[43] Fishbach A , Eyal T , Finkelstein SR . How positive and negative feedback motivate goal pursuit. Soc Personal Psychol Compass. 2010;4(8):517‐530.

[44] Bandura A . Self‐efficacy: toward a unifying theory of behavioral change. Psychol Rev. 1977;84(2):191‐215. 847061 10.1037//0033-295x.84.2.191

[45] Baretta D , Bondaronek P , Direito A , Steca P . Implementation of the goal‐setting components in popular physical activity apps: review and content analysis. Digit Health. 2019;5:2055207619862706. 31360535 10.1177/2055207619862706 PMC6637833

[46] Ferguson T , Olds T , Curtis R , et al. Effectiveness of wearable activity trackers to increase physical activity and improve health: a systematic review of systematic reviews and meta‐analyses. Lancet Digit Health. 2022;4(8):e615‐e626. 35868813 10.1016/S2589-7500(22)00111-X

[47] Gu X , Chen Y‐L , Jackson AW , Zhang T . Impact of a pedometer‐based goal‐setting intervention on children's motivation, motor competence, and physical activity in physical education. Phys Educ Sport Pedagog. 2018;23(1):54‐65. 10.1080/17408989.2017.1341475.

[48] Butcher Z , Fairclough S , Stratton G , Richardson D . The effect of feedback and information on children's pedometer step counts at school. Pediatr Exerc Sci. 2007;19(1):29‐38. 17554155 10.1123/pes.19.1.29

[49] Morgan CF , Pangrazi RP , Beighle A . Using pedometers to promote physical activity in physical education. J Phys Educ Recreat Dance. 2003;74(7):33‐38. 10.1080/07303084.2003.10609235.

[50] Emm‐Collison L , Cross R , Garcia Gonzalez M , Watson D , Foster C , Jago R . Children's voices in physical activity research: a qualitative review and synthesis of UK children's perspectives. Int J Env Res Public Health. 2022;19(7):3993. 35409676 10.3390/ijerph19073993 PMC8998303

[51] Liong GHE , Ridgers ND , Barnett LM . Associations between skill perceptions and young children's actual fundamental movement skills. Percept Mot Skills. 2015;120(2):591‐603. 10.2466/10.25.PMS.120v18x2. 25706343

[52] Nathan N , Elton B , Babic M , et al. Barriers and facilitators to the implementation of physical activity policies in schools: a systematic review. Prev Med. 2018;107:45‐53. 10.1016/j.ypmed.2017.11.012. 29155228

[53] Daly‐Smith A , Morris JL , Norris E , et al. Behaviours that prompt primary school teachers to adopt and implement physically active learning: a meta synthesis of qualitative evidence. Int J Behav Nutr Phys Act. 2021;18(1):151. 10.1186/s12966-021-01221-9. 34801039 PMC8605507

[54] Ellis DA , Piwek L . Failing to encourage physical activity with wearable technology: what next? J R Soc Med. 2018;111(9):310‐313. 10.1177/0141076818788856. 30032696 PMC6146340

[55] Lee VR , Drake J , Williamson K . Let's get physical: K‐12 students using wearable devices to obtain and learn about data from physical activities. TechTrends. 2015;59(4):46‐53. 10.1007/s11528-015-0870-x.

[56] Yu J , Xu T , Kelley C , Ruppert J , Roque R . Leveraging physical activities to support learning for young people via technologies: an examination of educational practices across the field. Rev Educ Res. 2024:00346543241248464. 10.3102/00346543241248464.

[57] Watchsted . OFSTED analysis comparisons. Available at: https://www.watchsted.com/analysis. Accessed November 22, 2023.

[58] UK Government . Academic year 2022/23: schools, pupils and their characteristics. Available at: https://explore‐education‐statistics.service.gov.uk/find‐statistics/school‐pupils‐and‐their‐characteristics. Accessed May 6, 2023.

