# Supplementary material for: Primary School Pupils' Perceptions and Experiences of Wearable Technologies
Source: J Sch Health. 2024 Nov 6;94(12):1119–28. doi: 10.1111/josh.13509 (PMC11693820; doi:10.1111/josh.13509)
Supplement: Supplementary file 1 — Table S1. The Consolidated Criteria for Reporting Qualitative research checklist table. [file JOSH-94-1119-s001.docx]

# Supplementary Materials

**The Consolidated Criteria for Reporting Qualitative research checklist table.**

| No | Item | Description |
| --- | --- | --- |
| Domain 1: Research team and reflexivity |  |  |
| Personal Characteristics |  |  |
| 1. | Interviewer/facilitator | GKW conducted focus groups |
| 2. | Credentials | MRes, working towards a PhD |
| 3. | Occupation | PhD researcher |
| 4. | Gender | Female |
| 5. | Experience and training | MRes in Health & Wellbeing, alongside additional doctoral training provided at GKW’s institution |
| Relationship with participants |  |  |
| 6. | Relationship established | GKW had previously conducted a different, but remote, research project in 3 out of the 5 schools in the previous academic year. Therefore, some, but not all, of the pupils had met GKW prior to this study. |
| 7. | Participant knowledge of the interviewer | The participants knew that the research was interested in understanding how physical activity could be improved in primary schools and 3 out of 5 schools had previously used wearable technologies within a research capacity. |
| 8. | Interviewer characteristics | No personal details shared with pupils; it was explained that the GKW was a researcher at their local University. |
| Domain 2: study design |  |  |
| Theoretical framework |  |  |
| 9. | Methodological orientation and Theory | Critical realism is the theoretical underpinning of GKW’s wider PhD research. Reflective thematic analysis was used to analyse the data. |
| Participant selection |  |  |
| 10. | Sampling | Participants were selected if they received parental/guardian consent. All pupils who received consent were included in the study. Schools were contacted if they had previously worked with GKW and local authority school contacts also disseminated recruitment information to invite new schools to participate. |
| 11. | Method of approach | Information sheets were send home to parents/ guardians. |
| 12. | Sample size | 41 |
| 13. | Non-participation | 1 pupil dropped out mid focus group as became agitated sat still and lost interest, all other pupils who received consent wished to participate for the full duration of focus groups. |
| Setting |  |  |
| 14. | Setting of data collection | In each pupils’ school environment in a quite classroom, or space appropriate for conducting the focus group. |
| 15. | Presence of non-participants | Just the researcher GKW present with a school staff member next door if children required them. |
| 16. | Description of sample | Year 5-6 pupils, in the South West of England, 19 girls, 22 boys. |
| Data collection |  |  |
| 17. | Interview guide | Yes, topic guide above, and study was piloted to check comprehension of questions with this age group. |
| 18. | Repeat interviews | No |
| 19. | Audio/visual recording | Audio recordings of the data were collected |
| 20. | Field notes | Yes, GKW made field notes at the end of each focus group |
| 21. | Duration | 39 minutes on average (range = ran between 31 to 45 minutes) |
| 22. | Data saturation | Reoccurring themes were coming up in field notes, but rather than grounding on data saturation a pragmatic approach was used. |
| 23. | Transcripts returned | Not applicable for this population and the time burdening nature of this approach. |
| Domain 3: analysis and findings |  |  |
| Data analysis |  |  |
| 24. | Number of data coders | 1 |
| 25. | Description of the coding tree | No, subthemes and codes discussed in text. |
| 26. | Derivation of themes | Derived from the data |
| 27. | Software | NVivo |
| 28. | Participant checking | No |
| Reporting |  |  |
| 29. | Quotations presented | Participant quotations presented with quotation identifiers |
| 30. | Data and findings consistent | ✓ |
| 31. | Clarity of major themes | ✓ |
| 32. | Clarity of minor themes | Some conflicting viewpoints highlighted |
